# Microarray Meta-Analysis Identifies Acute Lung Injury Biomarkers in Donor Lungs That Predict Development of Primary Graft Failure in Recipients

**DOI:** 10.1371/journal.pone.0045506

**Published:** 2012-10-12

**Authors:** Pingzhao Hu, Xinchen Wang, Jack J. Haitsma, Suleiman Furmli, Hussain Masoom, Mingyao Liu, Yumiko Imai, Arthur S. Slutsky, Joseph Beyene, Celia M. T. Greenwood, Claudia dos Santos

**Affiliations:** 1 The Centre for Applied Genomics, Hospital for Sick Children, Toronto, Ontario, Canada; 2 Keenan Research Center at the Li Ka Shing Knowledge Institute of St. Michael's Hospital, Interdepartmental Division of Critical Care, University of Toronto, Toronto, Ontario, Canada; 3 Thoracic Surgery Research Laboratory, Toronto General Research Institute, University Health Network, Toronto, Ontario, Canada; 4 Biological Informatics and Experimental Therapeutics Akita University Graduate School of Medicine, Akita City, Akita, Japan; 5 Program in Population Genomics, Department of Clinical Epidemiology and Biostatistics, McMaster University, Hamilton, Ontario, Canada; 6 Centre for Clinical Epidemiology, Lady Davis Institute and Department of Epidemiology, Biostatistics and Occupational Health, McGill University, Montreal, Quebec, Canada; University of Georgia, United States of America

## Abstract

**Objectives:**

To perform a meta-analysis of gene expression microarray data from animal studies of lung injury, and to identify an injury-specific gene expression signature capable of predicting the development of lung injury in humans.

**Methods:**

We performed a microarray meta-analysis using 77 microarray chips across six platforms, two species and different animal lung injury models exposed to lung injury with or/and without mechanical ventilation. Individual gene chips were classified and grouped based on the strategy used to induce lung injury. Effect size (change in gene expression) was calculated between non-injurious and injurious conditions comparing two main strategies to pool chips: (1) one-hit and (2) two-hit lung injury models. A random effects model was used to integrate individual effect sizes calculated from each experiment. Classification models were built using the gene expression signatures generated by the meta-analysis to predict the development of lung injury in human lung transplant recipients.

**Results:**

Two injury-specific lists of differentially expressed genes generated from our meta-analysis of lung injury models were validated using external data sets and prospective data from animal models of ventilator-induced lung injury (VILI). Pathway analysis of gene sets revealed that both new and previously implicated VILI-related pathways are enriched with differentially regulated genes. Classification model based on gene expression signatures identified in animal models of lung injury predicted development of primary graft failure (PGF) in lung transplant recipients with larger than 80% accuracy based upon injury profiles from transplant donors. We also found that better classifier performance can be achieved by using meta-analysis to identify differentially-expressed genes than using single study-based differential analysis.

**Conclusion:**

Taken together, our data suggests that microarray analysis of gene expression data allows for the detection of “injury" gene predictors that can classify lung injury samples and identify patients at risk for clinically relevant lung injury complications.

## Introduction

Acute lung injury (ALI) and acute respiratory distress syndrome (ARDS) are associated with significant morbidity and mortality (30–50%) [1]–[3]. Despite advances in supportive care, no therapies have shown benefit in large randomized clinical trials, other than the use of lung protective mechanical ventilation (MV) strategies. Exposure to repetitive cyclic stretch (CS) and/or over-inflation exacerbates injury. Reducing tidal volume (VT) improves survival. One reason for the lack of positive clinical trials may relate to our incomplete understanding of the pathogenesis of this syndrome. The paucity of ALI tissues for diagnostic and pathological studies, the high rate of intra-observer variability and the discrepancies between clinical and autopsy findings make it difficult to select patients for ongoing clinical trials and/or to identify clinically relevant classifiers of subgroups of patients for therapy. Moreover, interpreting mechanistic data from cell and animal models in the context of patients is a challenge. Accordingly, there is an urgent need to translate biologically relevant information to patients with lung injury.

To identify biomarkers [4], many studies have looked at the use of *a-priori* defined markers in pulmonary oedema fluid [5]–[7], blood [8]–[14] and urine [15]–[16] from ALI/ARDS patients. In parallel, genomic approaches have offered an unprecedented opportunity to perform “unsupervised" searches for novel biomolecular markers of injury. Experiments using microarray technology have identified individual gene expression markers of potential diagnostic and prognostic significance [17]–[18]. Our group has explored the global response to injury [19]–[20] and identified the presence of injury-specific expression profiles in comparable lung injury models. Here, genes that shared transcription profiles were biologically related, suggesting the information contained within expression profiles can help to identify and inform regarding mechanisms of ALI.

While individual microarray studies can be informative in identifying single genes [21] or significant biological pathways [22], it is still difficult to make direct comparisons between results obtained by different groups, since laboratory protocols, microarray platforms and analysis techniques differ appreciably. Most individual studies have relatively small sample sizes, and hence prediction models are prone to over-fitting, and are thus less robust and less generalizable; precluding the development of classification models that can be translated from animals to humans. Recent studies have shown that the systematic integration of gene expression data from multiple sources can increase statistical power for detecting differentially expressed genes while allowing for an assessment of heterogeneity, and may lead to more robust, reproducible and accurate predictions [23]–[26].

We used such an approach to conduct a cross-species and cross-platform meta-analysis of existing ALI-related microarray data. We approached the problem of data reproducibility by using a random-effects model to integrate the effect size of gene-specific expression changes in each individual experiment. We demonstrated the proof of concept by validating our approach using both animal and human external microarray data sets publicly available from the National Center for Biotechnology Information (NCBI) Gene Expression Omnibus (GEO). We used molecular injury expression profiles to classify human pulmonary cells exposed to various injury stimuli (cyclic stretch, Lipopolysaccharide (LPS) and tumour necrosis factor alpha (TNF-α) to their correct injury class and prospectively classify samples from a rat model of VILI. In addition, to demonstrate the clinical applicability of our approach, we used the meta-analysis results to develop a molecular classifier, a set of gene expression profiles, to detect clinically relevant lung injury in transplant donor lungs which would predict development of primary graft failure (PGF) in lung transplant recipients.

## Results

**Figure 1:**
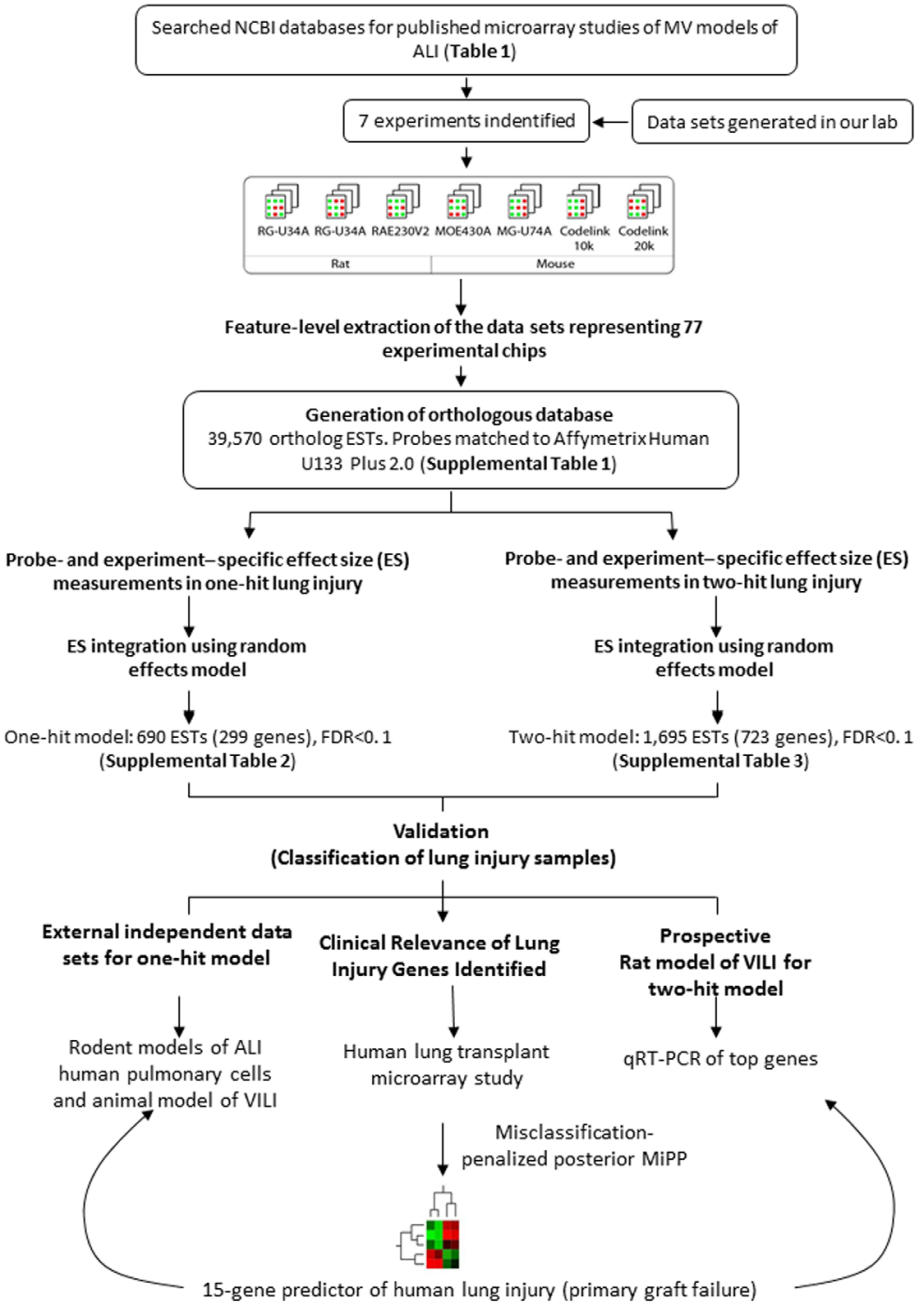
Flowchart of microarray meta-analysis and validation procedure. Differentially expressed genes were identified by meta-analysis of rat and mouse microarray expression data based on one-hit and two-hit animal lung injury models. Top genes were validated in an external data base on mouse model of VILI and in human cells exposed to cyclic stretch alone or in the presence of specific inflammatory mediators. Genes differentially expressed in one-hit model and two-hit models were used to build classifiers in donor lungs and predict lung injury and likelihood of primary graft failure in patients following lung transplantation.

We combined a total of 77 microarray chips across six platforms, two species and different animal lung injury models downloaded from NCBI/GEO and one unpublished experiment from our group (named as Rat_UP in **Table 1**). Since mechanical ventilation plays a critical immunomodulatory role in patients, we included only studies involving ventilated animal models of ALI (**Table 1**). Our goal was to determine the “effect size" of injury on gene expression (i.e. differential expression). Accordingly, individual gene chips from each of the studies were classified and grouped based on the original strategy used to induce lung injury (**Table 2**). We considered two main comparisons: the one-hit model which compared chips from animals exposed to “one-hit" models of overventilation lung injury: no ventilation (NV) or minimally injurious ventilation (low tidal volume, LV) vs. injurious ventilation (high tidal volume, HV); and the two-hit model which compared chips from animals exposed to “two-hit" models of lung injury, lung inflammation alone (Inf) vs. MV+Inf (two-hit model). **Figure 1** shows a schematic of the experimental design of our analyses. To compare between different microarray platforms, we generated a master database of orthologous probesets connecting individual platform specific probe identifiers (ID) to the Affymetrix Human U133 Plus 2.0 chip (See Materials and Methods). A total of 39,570 ortholog ESTs were included in the analysis (****).

**Table 1:** Summary of experiments included in microarray meta-analysis.

Experiments	Species	Platforms	Number of Chips	GEO Series Number	Injury mechanisms
GSE2411	Mouse	Affymetrix MOE430A	6	GSM45427 – 32	NV
			6	GSM45433 – 8	MV
			6	GSM45439 – 44	LPS
			6	GSM45445 – 50	MV+LPS
GSE2368	Rat	Affymetrix RG-U34A	2	GSM44768 – 9	NV
			2	GSM44770 – 1	MV
GSE2368	Mouse	Affymetrix MG-U74A	2	GSM44772 – 3	NV
			2	GSM44774 – 5	MV
Rat_UP*	Rat	Affymetrix RG-U34A	2	UP	MV
			2	UP	NV
			2	UP	HV+AA
			2	UP	MV+AA
			2	UP	AA
GSE2635	Mouse	Codelink Uniset Mouse-1 10 k	3	GSM50728–30	LV
			3	GSM50734–6	HV
GSE4215	Mouse	Codelink Uniset Mouse-1 10 k, 20 k	7	GSM96191–4 GSM96233 –6	LV
			5	GSM96195–7 GSM96237–41	LPS
			6	GSM96198–9 GSM96232 GSM96242–4	HV
			5	GSM96245–9	HV+LPS
GSE7041	Rat	Affymetrix RAE230V2	3	GSM162917–9	NV
			3	GSM162923–5	HV

Publicly available data were downloaded from NCBI GEO, and mechanism of injury used for animal hybridized to individual chips: Non-ventilated (NV), Mechanical ventilation (MV), high tidal volume (HV), low tidal volume (LV), lipopolysaccharide (LPS), acid aspiration (AA), and Unpublished (UP).

* Rat-UP: Unpublished rat gene expression data generated in this study.

**Table 2:** Group assignment of microarray chips.

Experiments	Platforms	Control group (non-or very mild lung injury samples)	Case group (lung injury samples)
**One-hit model of lung injury**			
GSE2411	Affymetrix MOE430A	NV (6 chips)	MV (6 chips)
GSE2368	Affymetrix RG-U34A	NV (2 chips)	MV (2 chips)
GSE2368	Affymetrix MG-U74A	NV (2 chips)	MV (2 chips)
Rat-UP	Affymetrix RG-U34A	NV (2 chips)	MV (2 chips)
GSE2635	Codelink Uniset Mouse-1 10 k	LV (3 chips)	HV (3 chips)
GSE4215	Codelink Uniset Mouse-1 10 k	LV (3 chips)	HV (3 chips)
GSE4215	Codelink Uniset Mouse-1 20 k	LV (4 chips)	HV (3 chips)
GSE7041	Affymetrix RAE230V2	NV (3 chips)	HV (3 chips)
**Two-hit model of lung injury**			
GSE2411	Affymetrix MOE430A	LPS (6 chips)	MV+LPS (6 chips)
Rat-UP	Affymetrix RG-U34A	AA (2 chips)	HV+AA (2 chips)
			MV+AA (2 chips)
GSE4215	Codelink Uniset Mouse-1 20 k	LPS (5 chips)	HV+LPS (5 chips)

All microarray chips were assigned, depending on the model of lung injury, to either of two comparisons: One-hit vs. Two-hit models of VILI. Microarray chips included in the meta-analysis (see **Table 1**) were classified according to the treatment strategies used to induce lung injury. For each comparison we determined the chips that would serve as either control or treatment based on the research question of interest. Non-ventilated (NV), mechanical ventilation (MV), high tidal volume (HV), low tidal volume (LV), lipopolysaccharide (LPS), and acid aspiration (AA).

### Meta-analysis of ALI-related models of lung injury

**Figure 2:**
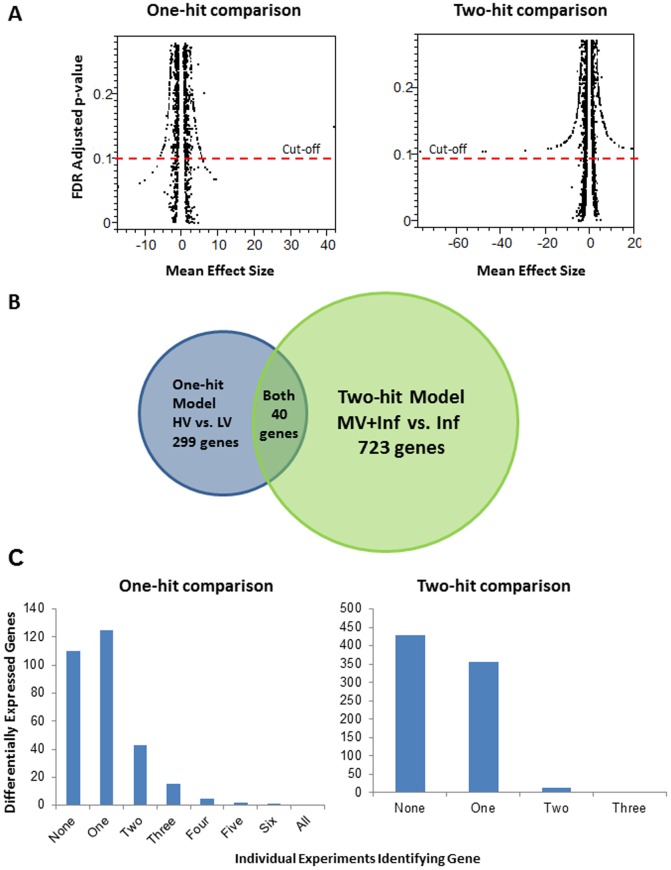
Identification of Acute Lung Injury Profiles. (**a**) **Relationship between adjusted pvalue and mean effect size (MSE).** (**b**) **Overlap of significantly expressed genes (FDR≤0.1) between one-hit and two-hit models of VILI**. Significantly altered probesets were matched between one-hit and two-hit models of VILI and collapsed to their corresponding genes using the annotation file for the Affymetrix U133 Plus 2.0 chip. (**c**) **Identification frequency of significantly expressed genes from one-hit and two-hit models of VILI**. Significantly expressed genes (FDR≤0.1) in the individual experiments were chosen using two-class unpaired SAM. Rat and mouse datasets from GSE2368 were considered one experiment, and normalized data provided in supplementary data was used for analysis. The frequency of the significantly expressed genes from meta-analysis of one-hit and two-hit models of VILI were evaluated by counting the times these genes were also found significantly expressed in individual experiment. Note: “All" means all the experiments used in meta-analysis; “None" means genes only found in meta-analysis but not in individual experiments.

For each comparison of the “one-hit" and “two-hit" models, the effect size (change in gene expression) was determined by the standardized mean difference for each gene in each individual experiment. We integrated the effect size across experiments using a random effects model [27]. The statistical significance of an overall change in gene expression across studies was provided by calculating the p-value corresponding to this *z*-statistic, and then estimating the false discovery rates (FDR) for each significance level. This takes into account the number of tests performed and corrects for multiple comparisons (adjusted p-value). We considered expressed sequence tags (ESTs) with an FDR≤0.1 to be differentially altered in each comparison. Despite large changes in expression (effect size) many genes were excluded on the basis of a high FDR (**Figure 2a**). A total of 690 and 1695 ESTs probe sets corresponding to 299 and 723 unique genes were identified as significantly changed for the one- and two-hit model comparisons respectively (**Tables S2 and S3**). The overlap between differentially regulated genes in the two models is shown in **Figure 2b**. **Tables 3** and **4** show the top 20 genes identified as significantly changed in the one- and two-hit models of lung injury. Thirty percent of all significant genes identified by the meta-analysis were not found to be differentially expressed when the individual studies were analyzed separately using SAM (significant analysis of microarray) (**Figure 2c**). This meta-analysis approach clearly identifies genes demonstrating consistent differential expression signals across studies. For example, among the top 20 genes, many of them show very stable expression patterns across the studies for either the one-hit model (**Figure S1a** or the two-hit model (**Figure S1b**). This strongly suggests that lung injury expression profiles of the most significant altered genes are most likely to be echoed across multiple platforms and species. It should be also noted that if a particular gene behaves completely differently in another species, it would (presumably) not rank highly in our meta-analysis frameworks.

**Table 3:** Top 20 genes identified by the microarray meta-analysis as differentially expressed in the one-hit model of VILI.

Gene Symbol	MES*	Adjusted p-value	No. of Exp**	Gene Title
TIPARP	4.74	0.00034	3	TCDD-inducible poly(ADP-ribose) polymerase
HK2	2.03	0.00045	8	Hexokinase 2
GADD45A	3.63	0.00139	8	Growth arrest and DNA-damage-inducible
CCRN4L	2.59	0.00203	6	CCR4 carbon catabolite repression 4-like (S. cerevisiae)
HOXB5	−1.97	0.00203	6	Homeobox B5
DLG3	−1.67	0.00203	8	Discs
IRX3	−1.97	0.00203	6	Iroquois homeobox protein 3
D4ST1	−2.16	0.00250	5	Dermatan 4 sulfotransferase 1
CD79A	−2.06	0.00251	5	CD79a molecule
CREM	3.01	0.00254	8	cAMP responsive element modulator
HSPB8	1.85	0.00254	6	Heat shock 22 kDa protein 8
CLDN7	1.58	0.00325	8	Claudin 7
ADCY6	−1.59	0.00359	8	Adenylate cyclase 6
SERTAD1	1.74	0.00359	6	SERTA domain containing 1
UBE2D3	1.75	0.00374	8	Ubiquitin-conjugating enzyme E2D 3 (UBC4/5 homolog)
ACTG1	1.70	0.03740	8	Actin, gamma 1
MCAM	1.70	0.00374	6	Melanoma cell adhesion molecule
TNFSF9	1.94	0.00374	5	tumor necrosis factor (ligand) superfamily
RBM17	1.77	0.00374	6	RNA binding motif protein 17
ST3GAL1	1.85	0.00398	5	ST3 beta-galactoside alpha-2

* MSE: mean effect size.

** The number of experiments the gene found.

**Table 4:** Top 20 genes identified by the microarray meta-analysis as differentially expressed in two-hit model of VILI.

Gene Symbol	MES	Adjusted p-value	No. of Exp**	Gene Title
CSAD	−4.56	0.00044	3	Cysteine sulfinic acid decarboxylase
PRX	−4.12	0.00044	3	Periaxin
TSPAN3	−3.84	0.00071	3	Tetraspanin 3
PCCA	−3.60	0.00111	3	Propionyl Coenzyme A carboxylase
FAH	−3.42	0.00143	3	Fumarylacetoacetate hydrolase
GSTA4	5.24	0.00143	3	Glutathione S-transferase A4
PLK2	3.24	0.00164	2	Polo-like kinase 2 (Drosophila)
ADORA2B	−3.25	0.00179	3	Adenosine A2b receptor
CD81	−4.66	0.00180	3	CD81 molecule
CBR4	−3.01	0.00250	2	Carbonyl reductase 4
NDUFAF3	−4.66	0.00250	3	NADH dehydrogenase (ubiquinone)
WBP1	3.01	0.00250	2	WW domain binding protein 1
BTG3	−4.45	0.00260	3	BTG family protein 3
PLVAP	−2.91	0.00260	2	Plasmalemma vesicle associated protein
PMP22	−2.86	0.00327	3	Peripheral myelin protein 22
NAGLU	−4.56	0.00473	1	N-acetylglucosaminidase
TENC1	−4.12	0.00473	3	Tensin like C1 domain containing phosphatase (tensin 2)
KCNJ8	−4.42	0.00489	3	Potassium inwardly-rectifying channel
LAMB1	4.42	0.0082367	3	Laminin
CAV1	4.59	0.0082367	3	Caveolin 1

### Functional enrichment analysis for differentially regulated genes

To assess the biological plausibility of the gene lists identified, we performed functional enrichment analysis in Ingenuity Knowledge Base (Ingenuity® Systems, www.ingenuity.com) for each gene-list separately (**Table 5**). The top functional enrichment for the one-hit comparison was for genes involved in developmental pathways. Of note, the one hit list was enriched for 11 genes encoding developmentally related transcription factors: Homeobox (B5, A5, B6, A3, D10), Iroquois 3 (Irx3), myeloid ecotropic viral integration site 1 homolog and site 1 homolog 2 (meis1 and meis2), pre-b-cell-leukemia transcription factor 2 (PBX2), lag1 longevity assurance homolog 4 (LASS4, s. cerevisiae), and pou domain, class 6, transcription factor 1 (POU6F1). In contrast, the two-hit list was enriched for genes involved in cellular motility, tetraspanin domain containing proteins (tetraspanin 3, 6, 7, 17, CD81 and CD9) and leukocyte related antigens (CD9/CD37/CD63). Both gene lists were enriched for genes involved in various canonical pathways previously implicated in VILI including ‘NF-κB signalling’, ‘IL-10 signalling’, ‘IL-6 signalling’ and ‘NRF-2 mediated oxidative stress response’ (**Table 6**).

**Table 5:** Predicted functional enrichment for genes identified by the one-hit and two-hit models of VILI.

One-hit Model			Two-hit Model		
Term	No. of Genes	P-value	Term	No. of Genes	P-value
Development	95	9.27E-14	Cellular Movement	79	6.47E-9
Cell Death	87	3.90E-13	Amino Acid Metabolism	58	9.55E-7
Immunological Disease	44	3.62E-9	Small Molecule Biochemistry	72	9.55E-7
Connective Tissue Disorders	29	6.98E-9	Cellular Growth and Proliferation	121	2.79E-6
Inflammatory Disease	40	6.98E-9	Cell Signalling	136	5.23E-6
Skeletal and Muscular Disorders	30	6.98E-9	Connective Tissue Development and Function	34	5.46E-6
Cellular Movement	49	3.45E-8	Drug Metabolism	17	6.84E-6
Hematological System Development and Function	37	3.45E-8	Cancer	114	7.18E-6
Immune Response	43	3.45E-8	Cell-To-Cell Signaling and Interaction	64	1.06E-5
Cancer	101	8.05E-8	Cellular Compromise	23	1.06E-5

Functional specific enrichment amongst differentially expressed genes identified using Ingenuity Pathway Analysis. Differential genes from one-hit and two-hit models were analysed separately.

**Table 6:** Top 10 canonical pathways enriched in one-hit and two-hit models of VILI.

One-hit Model			Two-hit Model		
Term	No. of Genes	P-value	Term	No. of Genes	P-value
Starch and Sucrose Metabolism	6	4.57E-5	LPS/IL-1 Mediated Inhibition of RXR Function	17	1.23E-6
NF-κB Signaling	8	5.50E-5	NRF2-mediated Oxidative Stress Response	15	1.35E-6
VEGF Signaling	6	9.33E-5	Integrin Signaling	18	6.16E-6
IL-10 Signaling	5	1.70E-4	Aryl Hydrocarbon Receptor Signaling	13	1.15E-5
Hepatic Cholestasis	7	2.40E-4	β-alanine Metabolism	10	4.47E-5
Galactose Metabolism	4	3.39E-4	Alanine and Aspartate Metabolism	9	4.47E-5
Integrin Signaling	8	4.57E-4	Xenobiotic Metabolism Signaling	17	6.03E-5
Toll-like Receptor Signaling	4	4.68E-4	Glutathione Metabolism	10	6.61E-5
IL-6 Signaling	5	8.13E-4	Butanoate Metabolism	10	1.55E-4
Hepatic Fibrosis/Hepatic Stellate Cell Activation	6	8.91E-4	Valine, Leucine and Isoleucine Degradation	9	8.91E-4

Canonical pathway specific enrichment amongst differentially expressed genes identified using Ingenuity Pathway Analysis. Differential genes from the one-hit and two-hit models were analysed separately.

### Validation of meta-analysis results

#### Validation using an External Microarray Data Set (Animal Models of ALI and VILI)

**Figure 3:**
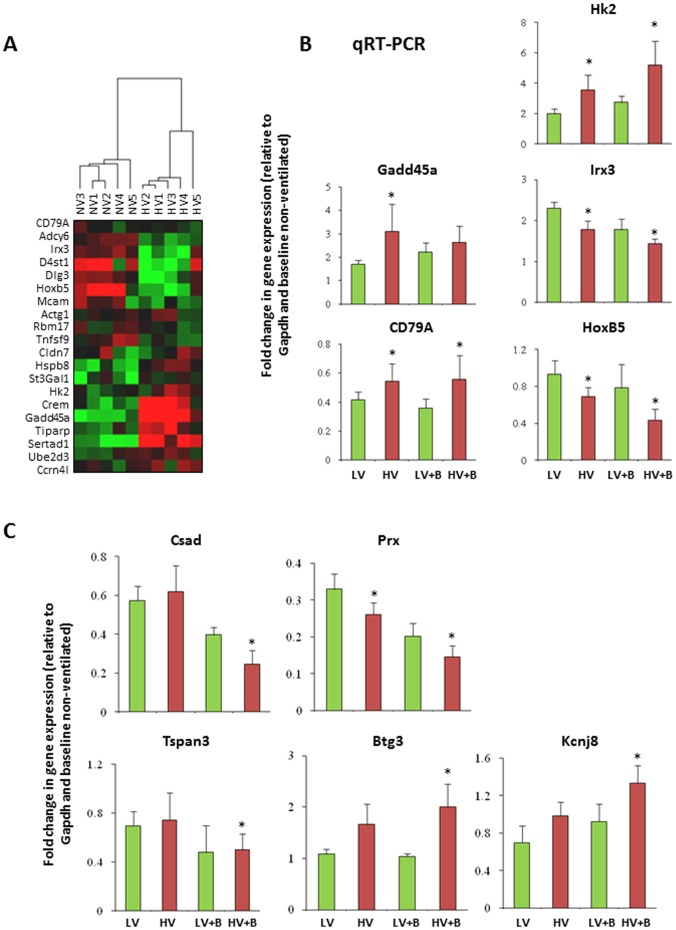
Validation using external animal lung injury samples. (a) Clustering of gene expression data of animal lung injury samples using top genes differentially expressed in one-hit model (NV or LV vs. HV). Top 20 genes were selected from one-hit model as ranked by lowest FDR to classify lung injury samples from an independent microarray experiment, not included in the meta-analysis. These genes were selected by mapping all differential genes with FDR< = 0.1 from the one-hit model to the external data set (GSE11434, **Table 7**). Unsupervised hierarchical clustering was used to cluster the data (*Red*, up-regulated in HV; *green*, down-regulated in HV). The top 20 genes from the meta-analysis identified group assignment correctly for all samples. Selected top genes identified in the one-hit model (**b**) and two-hit model (**c**) were confirmed by qRT-PCR in a rat model of lung injury.

We validated our gene lists using an external microarray data set that was not part of the meta-analysis. For this purpose, we ranked the genes identified in the one-hit model meta-analysis by adjusted p-value and empirically used the top 20 genes to classify an external data set of 10 whole lung (mice) injury samples down loaded from NCBI GEO (GSE11434 [28], **Table 7**). Normalized intensity values for individual gene probe sets were matched to our ortholog ID file (See Materials and Methods). Unsupervised hierarchical clustering correctly assigned all individual chips into their specific treatment groups (LV vs. HV ventilation) with 100% accuracy (**Figure 3a**). The distribution of prediction accuracies based on 1000 random sets of 20 genes with similar size is shown in (**Figure S2a**) and none of the 1000 random sets gave 100% accuracy.

**Table 7:** Summary of studies used for validation.

Experiments	Species	Platform	# of Chips	Experiment Details
GSE11434	Mouse	ffymetrix MOE430V2	10	Mice were anaesthetized. Saline (0.25 mL) was given every hour ip. Mice were ventilated with an initial peak airway pressure of 20 cmH2O approximating a tidal volume of 20 mL/kg and without end-expiratory pressure. Ventilation was continued for 3 h. Tidal volume was not adjusted. Control mice were treated identically, but were not mechanically ventilated (i.e. breathed spontaneously). There were 5 biological replicates in each group.
GSE16650	Human	Affymetrix U133plus2	12	Bronchial epithelial distal airway small cells (BEAS2b) were randomized to 6 treatment groups (i) static control, (ii) cyclic stretch (22% elongation, 30 cycles/min), LPS (1 µg/µL), TNF (20 ng/µL), LPS+cyclic stretch and TNF+cyclic stretch
Human_UP*	Human	ffymetrix U133plus2	12	Small Airway Epithelial Cells (SAEC) were randomized to 6 treatment groups (i) static control, (ii) cyclic stretch (22% elongation, 30 cycles/min), LPS (1 µg/µL), TNF (20 ng/µL), LPS+cyclic stretch and TNF+cyclic stretch
GSE8021	Human	ffymetrix U133A 2.0	50	50 human donor lung samples were divided into two groups - those that developed PGD after transplantation (PGD positive) and those that did not (PGD negative)

* Human-UP: Unpublished human gene expression data generated in this study.

#### Prospective Validation using Rat Model of Acute Lung plus VILI

To prospectively validate the top genes identified by the meta-analysis, we conducted new experiments in the lab using a well established clinically relevant model of pneumonia exposed to injurious or non-injurious MV. Sprague Dawley rats were randomized to intra-tracheal instillation of *Klebsiella pneumoniae or equal volume saline*. After 24 hrs rats were re-randomized to receive MV with either non-injurious LV (6 ml/kg and 5 cm H2O of PEEP) or injurious HV (12 ml/kg without PEEP) ventilation for 3 h [13]–[15]. At the completion of the experiment, total RNA was extracted from whole lungs and the expression of the top 20 genes identified through the meta-analysis (one-hit model in **Figure 3b** and two-hit model in **Figure 3c**) were successfully confirmed prospectively by qRT-PCR.

#### Validation in Human Lung Cell Line and Primary Pulmonary Cells

**Figure 4:**
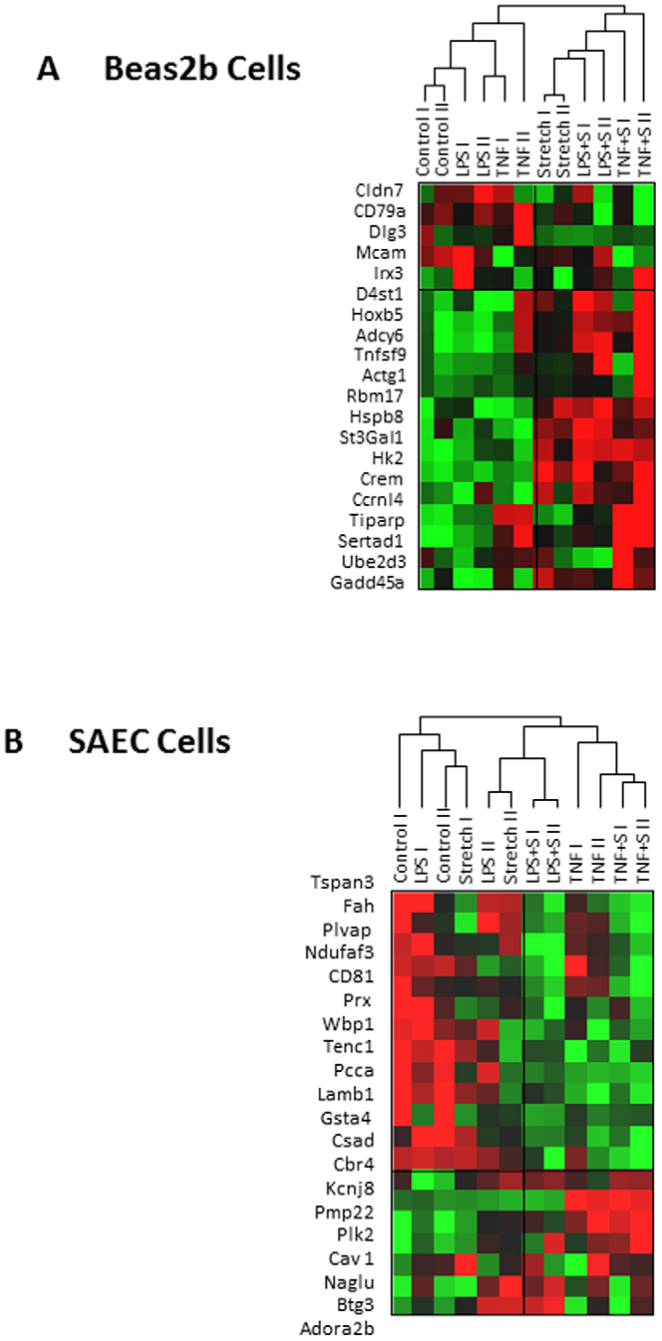
Validation using human pulmonary cells exposed to cyclic stretch with or without combined exposure with two inflammatory insults. Top 20 significantly expressed genes from one-hit model or BEAS2b (a) and two-hit model or SAEC (b) were mapped to the external gene expression data of human pulmonary cells. Unsupervised hierarchical clustering was used to cluster the data (Red, up-regulated in HV; green, down-regulated in HV). The top 20 genes from the meta-analysis identified group assignment correctly for all samples.

Our lab has previously profiled differential gene expression in human bronchial small airway epithelial cells (BEAS2b) in response to cyclic stretch alone, or in combination with two distinct inflammatory insults - lipopolysaccharide (LPS) or tumour necrosis factor alpha (TNFα) (GSE16650 [29]). An identical experimental design (one-hit, cyclic stretch alone and two-hit, cyclic stretch plus inflammation induced with either LPS or TNF-α) has been performed in primary small airway epithelial cells (SAEC, unpublished data from our lab named as Human_UP in **Table 7**). We performed unsupervised clustering of gene expression data from both BEAS2b and SAEC using the top 20 genes from the meta-analysis. The gene list generated by the one-hit model comparison correctly grouped cells that had been exposed to cyclic stretch with 100% accuracy (**Figure 4a**). The distribution of prediction accuracies based on 1000 random sets of 20 genes with similar size is shown in (**Figure S2b**), and again, none of the 1000 random gene sets demonstrated 100% prediction accuracy. The top 20 genes from the two-hit model (**Figure 4b**) correctly classified chips from cells exposed to two-hits (cyclic stretch plus LPS or TNFα) but classified the TNFα alone group with the two-hit chips. The dendogram suggests TNF-α is a powerful transcriptional stimulus generating greater transcriptional similarity than that between other competing injury stimuli.

We have demonstrated that selecting top differentially-expressed genes via meta-analysis leads to a replicable set able to classify both human and rat cells to the correct injury model. In contrast, we also performed computational validation using the top 20 genes selected from the one-hit model in a single study (GSE2411, **Table 2**), one of the studies in the meta-analysis. These genes were used to classify lung injury samples from the same independent microarray experiment that was used above, (GSE11434, **Table 7**) (**Figure S3a**), and also to classify human bronchial epithelial distal airway small cells from the (BEAS2b) microarray experiment (GSE16650, **Table 7**), which was also used above (**Figure S3b**). The top 20 genes from the differential analysis of the individual study used in meta-analysis can identify group assignment correctly for all independent animal samples (**Figure S3a**), but cannot classify the human samples correctly (**Figure S3b**). Therefore, we suggest that the top genes selected from the meta-analysis approach have better power to predict human lung injury samples than those selected from a single study. For other single studies in **Table 2**, we did not perform the above analysis because they have too small sample size [2]–[4] to be analysed along.

### Developing a molecular classifier of clinically relevant ALI

To determine whether our approach in developing a ‘gene-gene’ meta-analysis could generate clinically informative data that could be translated to humans, we used genes from the meta-analysis to classify human samples from patients who we thought could have lung injury. Frozen lung tissue samples from ALI/ARDS patients are very difficult to obtain. We used available gene expression data generated from transplanted donor lungs. The microarray chips were downloaded from NCBI GEO (GSE8021 [30] in **Table 7**). In this experiment, total RNA from 50 donor lungs were collected and hybridized to Affymetrix Human U133 Plus 2.0 chips. Gene expression profiles from donors were used to predict the advent of primary graft failure (PGF) in transplant recipients. PGF is a devastating form of ALI that mimics ARDS clinically. In addition, most donors receive short periods (usually days) of MV. Probe set IDs were matched to our ortholog database as described. We hypothesized that if the differentially expressed genes identified by the meta-analysis were truly representative of an acute “injury" profile, then this profile would be “transplanted" to the recipients and be associated with the development of PGF. More importantly, healthy patients, who do not develop PGF should not develop a transcriptional profile consistent with ALI.

We used a supervised misclassification-penalized posteriors (MiPP) [31] classification algorithm to develop a molecular predictor to classify clinically relevant ALI (See Materials and Methods for detail). As a first step, we evaluated the ability of the two differential gene lists from meta-analysis (**Table S2** for one-hit model and **Table S3** for two-hit model) to predict PGF. We split the dataset into a training group (33 samples) and a test group (17 samples) and used the MiPP algorithm to sequentially select genes one at a time using linear discriminant analysis (LDA) and 5-fold cross-validation in the training group. Through 20 randomly partitions of the 50 samples into training and test groups, differentially regulated genes from the two-hit model were able to predict PGF with an average accuracy of 82.4% in the training group and 71.3% in the test group. In contrast, genes from the one-hit model predicted PGF with an average accuracy of 88.9% in the training group and 77.6% in the test group. Therefore, both of our selected gene lists gave fairly good predictions of PGF. However, we also found that the gene list obtained from the one-hit model was slightly more informative in accurately classifying patients with and without PGF

**Figure 5:**
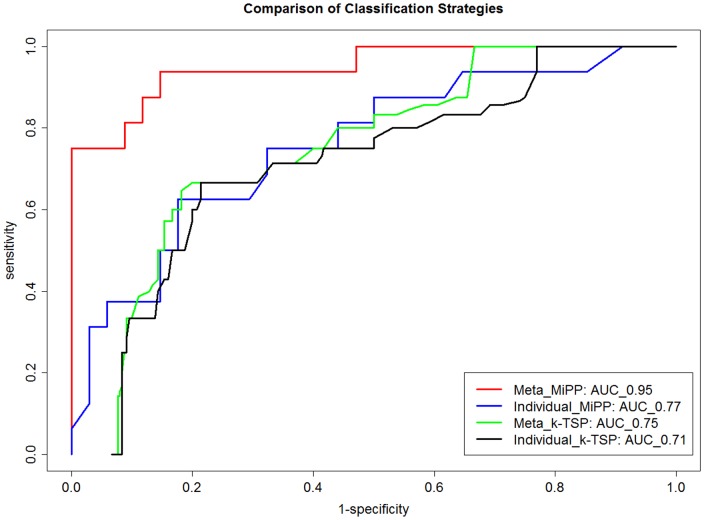
Comparison of classification strategies. The receiver operating characteristic (ROC) of PGF and GOOD samples using four classification strategies: (a) **Meta_MiPP**: Starting from the 690 significant probesets (FDR< = 0.1) selected by meta-analysis we proposed in the study. An optimal 15_gene list from the 690 probesets was selected from MiPP classifier. ROC curve is drawn based on the five-fold cross-validation of the 50 human donor lung samples (GSE8021, **Table 7**) using the 15-gene prediction model. (b) **Individual_MiPP**: Top 690 probesets were selected from one-hit model in an individual study (GSE2411, **Table 2**) based on effect size. The reason for selecting this study is that it has the largest sample size. The performance of the MiPP classifier built on 25-genes optimally chosen from the 690 gene list in the single study is evaluated by ROC using five-fold cross-validation. (3) **Meta_k-TSP**: Starting from the 690 significant probesets selected by meta-analysis, then an optimal 9_gene pairs from the 690 probesets was selected from k-TSP classifier. The k (k = 9) and ROC curve are based on the five-fold cross-validation. (4) **Individual_k-TSP**: Top 690 probesets were selected from one-hit model in an individual study (GSE2411, **Table 2**). An optimal 1_gene pairs from the 690 probesets was selected from k-TSP classifier. The k (k = 1) and ROC curve are based on the five-fold cross-validation.

**Figure 6:**
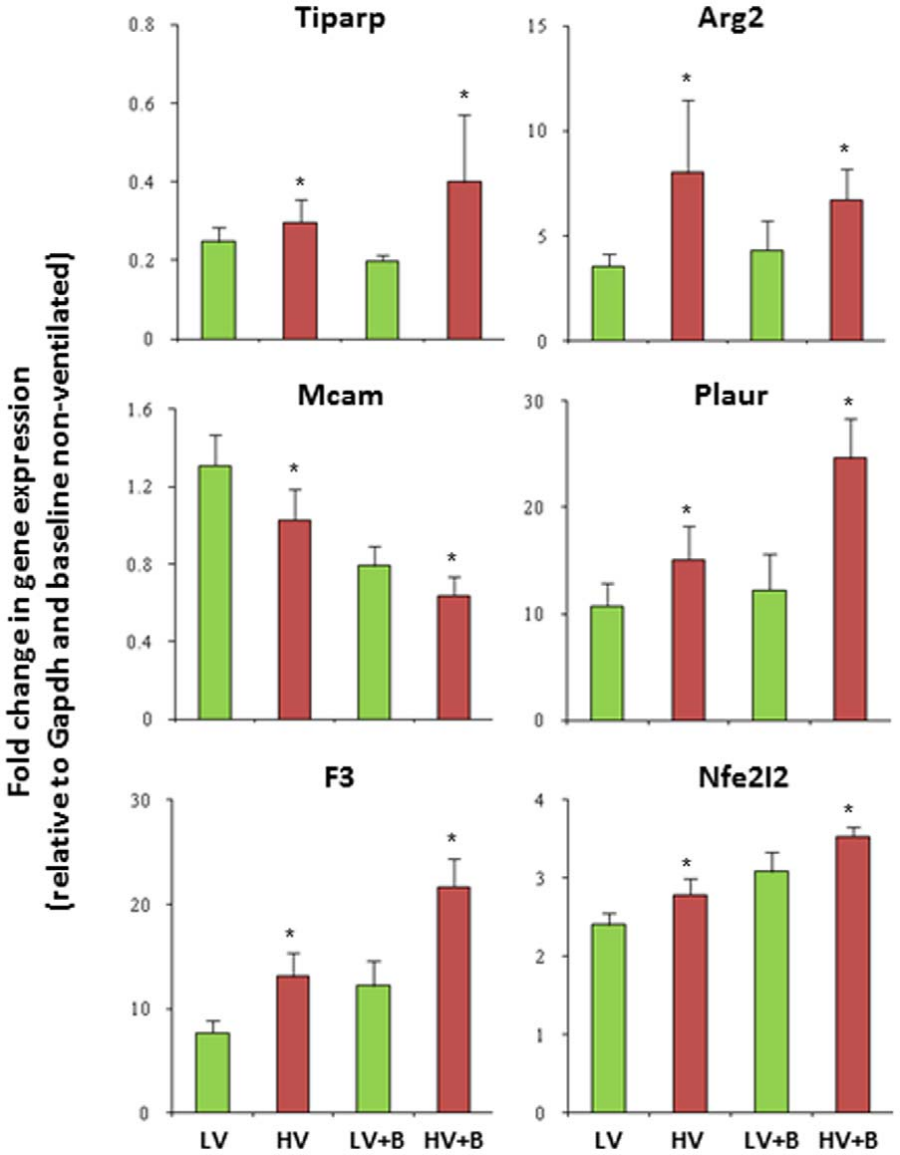
Validation of the genes used in the prediction model by qRT-PCR. qRT-PCR of selected genes. The gene expression was normalized against GAPDH. The changes in gene expression are expressed as fold change relative to GAPDH and the baseline sample from non-ventilation samples. Data are presented as means± SEMs (n = 3), * p<0.05 between non-ventilated and ventilated groups. TCDD-inducible poly(ADP-ribose) polymerase (Tiparp); arginase, type II (Arg2), melanoma cell adhesion molecule (Mcam), plasminogen activator, urokinase receptor (Plaur), coagulation factor III (F3, thromboplastin, tissue factor), and nuclear factor, erythroid derived 2, like 2 (Nfe2l2).

We further performed a five-fold cross-validation on the full 50-sample dataset using the differential genes identified from meta-analysis in the one-hit model (**Table S2**). **Figure 5** shows the receiver operating characteristic (ROC) and the area under the curve (AUC) of the prediction model with an optimal set of 15-genes chosen in the MiPP classification procedure (See Materials and Methods). The analysis procedure is named as Meta-MiPP. The accuracy was 86% with a sensitivity of 75% and a specificity of 91.2% for the 50-sample dataset, compared to 57.7% using P∶F ratios (the ratio of arterial oxygen concentration to the fraction of inspired oxygen) alone to predict PGF. The change in gene expression profile of 6 genes used in all of the 5-fold cross-validation experiments was determined by qRT-PCR in lung tissues from rats used in the prospective *Klebsiella pneumonia* plus VILI (**Figure 6**).

We also performed a five-fold cross-validation on the full 50-sample dataset using the top 690 differential probesets identified from the individual study with the largest sample size (GSE2411, **Table 2**) in the one-hit model. We also used the MiPP classification procedure. The analysis procedure is named as Individual-MiPP. Its receiver operating characteristic (ROC), and the area under the curve (AUC) of the prediction model with an optimal set of 25-genes is also shown in **Figure 5**. Comparing with the performance of the MiPP predictor built on 15-genes optimally selected from the differential gene list in one-hit model (**Table S2**), the performance of the MiPP classifier built on 25-genes optimally chosen from the differential gene list in the single study is worse (accuracy 72%, sensitivity 50%, specificity 82%).

We also compare classification performance using a second predictive model, the k-top scoring pair (k-TSP) classification algorithm [32]. To make a fair comparison, we used the same 690 probesets in **Table S2** used in Meta-MiPP and the same top 690 differential probesets identified from the individual study with the largest sample size (GSE2411, **Table 2**) in the one-hit model and used in Individual-MiPP. The k-TSP algorithm based on the two gene lists are named as Meta_k-TSP and Individual_k-TSP, respectively. The five-fold cross-validation procedure identified the optimal k to be 9 and 2 (or 9-gene pairs and 2-gene pairs) for Meta_k-TSP and Individual_k-TSP, respectively. As shown in **Figure 5**, Meta_k-TSP classifier has a larger AUC value than Individual_k-TSP, suggesting the gene signatures identified from our meta-analysis framework has improved classification performance over those identified from individual study. It should be noted that the major focus of our study is on integrating microarray data sets across platforms and species to identify biomarkers for predicting development of primary graft failure in recipients, rather than using gene signatures identified from individual studies. We used existing classification methods (MiPP and k-top scoring pairs) to perform the prediction. In this sense, we are not claiming one classification method (such as MiPP) is better than another method (such as k-top scoring pair).

## Discussion

Whole genome profiling using microarray technology has emerged as an ‘unsupervised’ strategy to identify potential candidate “disease" informative genes. Differential expression of genes in response to a particular stimulus is used as an indicator of molecular phenotype(s). An extension of this method relies on the hypothesis that co-expression of genes in response to a specific stimulus - across species - could also be exploited. In the case of lung injury, this commonality might relate to unsuspected evolutionarily conserved responses to lung injury. In this study, we provide a method for gene-by-gene comparison and analysis of microarray datasets originating from multiple species and injury models of ALI. Our main goal was to capitalize on existing electronic data, identify biologically and statistically robust ways to integrate critical information contained in microarray studies, and demonstrate that integration of experimental data can be a valuable method to identify biologically relevant signatures of lung injury that may have an impact on patient care. Our results show that the genes selected from our meta-analysis have potential clinical application in developing a predictor model for lung injury.

A key biological concept used in this study to integrate data from different experiments relies on the idea that an individual “disease phenotype" (lung injury) is comprised of the sum of cell and organ-specific, developmental stage, and metabolism related changes in gene expression. Genome-wide gene regulatory networks govern this behaviour. Theoretical studies of complex networks suggest that these can exhibit ordered (stable) dynamics, raising the possibility that molecular phenotypes of illness may represent high-dimensional attractor states that can be identified by whole genome analysis of expression patterns. To demonstrate proof of concept, we show that the relevant biological pathways predicted by the meta-analysis include those previously implicated in ALI and VILI, such as NF-κB and IL-6 signalling, lending support to the validity of our meta-analysis model [33]. We also identified many functional groups related to tissue development and cellular growth, two classifications which are well-linked to the pathogenesis of lung injury. Here, we note that many genes from the homeobox family, including HOXA5, HOXB5, and HOXB6, which have not been implicated in the tissue remodelling process of lung injury, were found significantly down-regulated in our study. In particular, other studies have shown that HOXA5 has normal expression levels in adult human lungs, and hypothesize that it plays a prominent role in postnatal lung homeostasis. HOXA5^−^/^−^ mice exhibit respiratory distress syndrome-like symptoms, and show decreased expression levels of lung surfactant-related proteins [34], suggesting that down-regulation of homeobox genes may be involved in the pathogenesis of VILI, and are intriguing subjects for the future study of lung injury.

Various groups [35]–[36], including our own [20], have hypothesized that acute injury profiles are “echoed" across species and models. The unique aspect of our study is the methodology used to detect such profiles. Our approach substantially increases sample size, reducing the effects of noise, thereby allowing identification of biologically meaningful genes and patterns with a higher statistical level of confidence. To demonstrate the validity of our methodology, we used genes identified by the meta-analysis to classify lung injury samples from both animals and human cells. Two important observations were made: First, genes selected from the meta-analysis could identify injury phenotypes with a probability significantly better than random chance alone; and second, the “one-hit" gene list was able to correctly classify samples in all validation models more consistently and accurately. One potential explanation for this is that while the appearance of inflammation-related expression profiles may be transient because of fluctuations in cellular and humoral mediators, the effects of cyclic mechanical forces on lung parenchyma are more consistent and repetitive. This allowed us to detect similar response profiles in a human pulmonary cell line, primary lung cells, animals and humans exposed to cyclic stretch.

We chose to demonstrate the performance of our model on human lung transplant patients with PGF because this complication can manifest with symptoms similar to ARDS and (contrary to ARDS) microarray data from lung tissues are available. We only had access to lungs that were actually transplanted. Severely injured lungs were not included in the study because they are rejected based on clinical profiles. This biased our study towards a more “benign" profile - thus likely leading to less pronounced molecular differences (effect-size) between donor lungs that may or may not develop PGF down the road. Another important limitation is that we did not have access to the ventilation history of patients. We contacted the authors to obtain details about ventilation protocols but this information had not been recorded. Notwithstanding, we were able to predict development of PGF in lung transplant recipients based upon injury profiles within lungs from transplant donors. The accuracy of our prediction was 86% with a sensitivity of 75% and a specificity of 91.2% for the 50-sample dataset, compared to 57.7% using P∶F ratios alone. Our data suggests an important link between lung injury (in donors) and outcomes post-transplantation (recipients). Future prospective studies will address the relationship between ventilation history and development of acute injury gene expression profiles in donors and development of PGF in recipients. Future studies will also look at the utility of the lung injury profile generated by the meta-analysis to classify lungs that have a “severe" injury profile and should therefore be excluded from transplantation.

In our study, the MiPP gene signatures include 15 genes: TMEM134, PIGQ, CH25H, ARPC3, MCAM, TIPARP, F3, AP4S1, C14orf133, PLAUR, NFE2L2, CYR61, ARG2, TMEM183A and GLRX. A number of these genes have been found to be ‘biologically’ relevant in various in-vitro and in vivo models of sepsis and ALI/ARDS. TIPARP, a member of the PARP family of genes, is responsible for maintaining genomic stability by sensing and repairing DNA damage. In the present study, TIPARP expression was significantly up-regulated in lung tissues from rats exposed to HV, supporting the importance of the PARP family of genes in DNA repair and suggesting a novel role for TIPARP in the development of biotrauma. The master transcription factor - nuclear respiratory factor-2 (Nrf2 or NFE2L2) binds to the antioxidant response element on a variety of anti-oxidant related genes thereby coordinating the response to oxidative stress. Nrf2 deficient mice are more sensitive to ALI and VILI. In patients, mutations in Nrf2 are associated with susceptibility to ALI/ARDS [37]. Plasminogen activator urokinase receptor (PLAUR) and tissue factor (F3, coagulation factor III, or thromboplastin) are up-regulated at the mRNA and protein level in LPS and cyclic stretch induced lung injury [38]–[39]. A series of clinical studies in humans have evaluated the clinical utility of anti-tissue factor treatment for sepsis and ALI/ARDS [40]. In contrast, although multiple studies have found the anti-oxidant gene, glutaredoxin (GLRX), to be implicated in lung injury, mutations in this gene have not resulted in susceptibility to lung injury [41]. Arginase catalyzes the hydrolysis of arginine to ornithine and urea – it is a fundamental enzyme in nitric oxide metabolism. At least two isoforms of mammalian arginase exists (types I and II) which differ in their function and localization. The type II isoform is located in the mitochondria and is expressed in extra-hepatic tissues. Arginase 2 (ARG2) variations are known to be associated with asthma, asthma severity and beta2 agonist and steroid response [42]. The matri-cellular proteins cysteine-rich, angiogenic-inducer, 61 (CYR61) has been implicated as a potential biomarker for ALI/ARDS/VILI and Fas-induced lung fibrosis. Moreover, this gene is regulated in sepsis-induced multiple organ failure [43]. Melanoma cell adhesion molecule (MCAM) is a critical molecule involved in adhesion, cellular migration, metastasis and trafficking. Although the gene for MCAM has been found to be upregulated in most models of lung stretch (animal and cell based), to date no studies have looked at its role in lung injury [20]. N-acetylglucosaminyl transferase component Gpi1 (PIGQ) is involved in the first step in glycosylphosphatidylinositol (GPI)-anchor biosynthesis. The GPI-anchor is a glycolipid found on many blood cells and serves to anchor proteins to the cell surface. ARPC3 (actin related protein 2/3 complex, subunit 3) encodes one of seven subunits of the human Arp2/3 protein complex - a key regulator of the actin cytoskeleton. The Arp2/3 protein complex has been implicated in the control of actin polymerization in cells and is conserved through evolution. C14orf133 encodes for a vacuolar protein sorting-associated protein 33B (VPS33B) interacting protein. This is a known apical-basolateral polarity regulator that plays a role in lysosomal trafficking. The gene product may play a role in epithelial polarization through stabilization of apical membrane protein content, and although the role of this gene has not been explored it is biologically plausible that it may play a role in ALI/ARDS. In contrast, the remaining genes are not known to play a role in ALI/ARDS and represent novel genes identified by the meta-analysis. Two of the genes identified, TMEM134 and TMEM183A, are members of a family of hypothetical transmembrane proteins, none of which have any known function.

Traditionally, meta-analyses of microarray datasets are used to take advantage of an increased sample size, allowing for the identification of a more statistically robust set of genes altered between treatment and control conditions. In this study, we expand on this principle by conducting a gene-by-gene comparison of data from multiple microarray platforms that also leverages information from multiple species. Since *in vivo* studies of ALI and other clinical complications are normally conducted in model organisms, applying the biological results to human settings can be challenging. A cross-species meta-analysis as performed here will preferentially select *against* genes differentially expressed in only one species, and instead prefer an evolutionarily conserved set of genes that can better translate to clinical settings. As well, we incorporate data taken from a variety of injury models. Notably, studies GSE2411, GSE2368, and GSE7041 tested an *in vivo* model of positive pressure ventilation, whilst GSE2635 and GSE4215 applied an *ex vivo* model with negative pressure ventilation. Inclusion of the *ex vivo* model lessens the signal contributions of non-epithelial cells such as neutrophils, contributing to a more specific set of genes identified.

It is worth noting that there will be many differences between data sets when integrating microarray data sets. In addition to the usual batch and lab effects, there will also be differences by platform and species. Raw gene expression levels may not be comparable across studies. Our meta-analysis was careful to combine standardized effect sizes, and we assume therefore that the standardized effects have generalized interpretability. Ideally, it would be possible to measure expression in a standardized way across many studies, however this information and such data sets were not available. It would be interesting to investigate if a meta-analytic gene selection strategy performs even better when platform and laboratory effects are known and controlled.

Ours is the first study to formally conduct a gene-gene meta-analysis of ALI related microarray data. Similar studies reviewing the effects of VILI across species were conducted by Wurfel and Grigoryev *et al.* [35]–[36]. However, we note that Wurfel's review incorporates only four independent VILI-related experiments and more importantly, genes were classified as differentially expressed only if they were significantly altered in at least two of the substituent experiments. Instead, our methodology integrates all gene expression values within all studies before calculating the mean effect sizes and FDR. Thus, our method is able to identify genes with small but consistent differential expression, while Wurfel's approach can only filter an existing set of significant genes. Grigoryev *et al.*'s study is similar in that it attempts to identify VILI-related genes orthologous between mice and rats. However Grigoryev *et al.* evaluates data from species separately before choosing genes commonly up or down-regulated in both datasets whereas our methodology integrates all datasets together when calculating mean effect sizes and FDR, allowing for identification of more robust biomarkers. As well, our meta-analysis is unique in that in our approach we vastly increase the sample size tested from 8 microarray chips to 77.

In this study we made the choice to not weight experiments based on sample size. This choice was made in order to give equal treatment to the different experimental protocols used in constituent studies, and not bias results towards one specific experiment. However, we noticed that the consequent incorporation of an extra dataset with no replicates worsened the meta-analysis results due to the addition of extra noise. Therefore, the quality of output from the meta-analysis will depend upon the sample sizes of constituent experiments, and care must be taken in future experiments to filter experiments for data quality. As well, the integrative nature of our meta-analysis invites some difficulties when choosing datasets to include. We attempted to evaluate the time-dependent biological response to VILI, but lacked sufficient data points to do so and hence, biologically interesting genes with a time-dependant pattern of expression were likely lost in the analysis. As more microarray data sets are uploaded into public repositories we expect time-series analyses to become more feasible.

Our one-hit model investigates the global effects of injurious HV on pulmonary gene expression. Two-hit model emulates the clinical situation where a patient is admitted with a pre-existing lung injury (i.e. pneumonia/acid aspiration) and is then ventilated; therefore it explores the combined effects of ventilation and inflammatory injury. Conceptually it is possible to imagine how this approach may yield important information that may enable clinicians to differentiate different forms of lung injury and tailor appropriate treatment strategies. In the future, prospective validation and refining of molecular markers of lung injury may yield important clues as to the pathogenesis, prognosis and clinical response to therapy in patients with ALI.

## Conclusions

We have developed a novel procedure for the meta-analysis of microarray data originating from multiple species and platforms. The results presented here demonstrate that this approach can yield biologically relevant data, thus identifying interesting candidate genes for future research, and is relevant to other external datasets, suggesting the results are not study-specific. In addition, this methodology can be generalized to other diseases and can be applied to the extensive data stored in online microarray repositories, thus allowing for in-depth re-analyses of previously published microarray experiments which are capable of identifying biologically interesting, but previously excluded genes. The promise of “fast" genomic solutions to major clinical problems has by enlarge gone unfulfilled in the field of ALI and ARDS. The challenge however is to build on knowledge acquired, using innovative approaches, and push limits of the technology with a clear sense of purpose – improved patient care.

## Materials and Methods

### Studies included in the meta-analysis

Animal and human studies of VILI using microarrays/gene chips published available on the National Center for Biotechnology Information (NCBI) Gene Expression Omnibus (GEO) database (http://www.ncbi.nlm.nih.gov/geo/) were reviewed for inclusion and obtained from our own lab. We included five publicly available studies (GSE2411, GSE4215, GSE2635, GSE2368, GSE7041), representing seven separate experiments for the meta-analysis. As well, we included microarray data from an additional unpublished study with 12 chips from our own group, bringing the final number of chips used in the meta-analysis to 77. The basic criteria to include the experiments in our meta-analysis are that (1) experiments were performed to compare differential expression in whole lung tissues; (2) the ventilation time was in a 2–5 hour window; (3) multiple biological replicates are available within groups. A summary of the studies included in the meta-analysis is shown in **Table 1**.

### Pre-processing raw gene expression data

For all studies using Affymetrix chips we downloaded the raw data files (.cel files). The *affy* Bioconductor R package was used to preprocess all raw data files. Extraction of probe level data, background correction, normalization using robust multi-array average (RMA) algorithm [44] was performed for each individual experiment to summarize probeset-levels of expression. Because the data using Codelink chips came from the same laboratory and were analyzed similarly we were able to use the normalized data files downloaded from NCBI GEO directly.

### Construction of Ortholog Database

To standardize the annotation data between species and microarray platforms, probes from each chip used in the meta-analysis were matched to the human Affymetrix U133 Plus 2.0 microarray chip. Probe sets were matched based on probe sequences identity as available from the annotation and probe sequence files downloaded from Affymetrix (http://www.affymetrix.com/index.affx) and Applied Biosystems Arrays (http://docs.appliedbiosystems.com/pebiodocs/00113304.pdf), using Resourcerer [45] (http://compbio.dfci.harvard.edu/tgi/), Madgene (http://www.madtools.org), or by Basic Local Alignment Search Tool (BLAST) search. In Resourcerer and Madgene, expressed sequence tags (ESTs) are identified using the Eukaryotic Gene Ortholog (EGO) database (http://compbio.dfci.harvard.edu/tgi/ego/). Sequence similarities are identified by stringent pair-wise comparison between tentative consensus sequences available from the Institute for Genomic Research (TIGR) Gene Indices (http://www.tigr.org/tdb/tgi). BLAST finds regions of similarity between biological sequences (http://blast.ncbi.nlm.nih.gov/Blast.cgi). We searched them using the nucleotide database query for sequence similarities between ESTs. TIGR Orthologous Gene Alignment (TOGA; http://www.tigr.org/tdb/toga/toga.shtml) database was used to provide a cross-reference between fully and partially sequenced eukaryotic transcribed sequences. To identify functional orthologs we further manually curated the ortholog database by searching HUGO Gene Nomenclature Committee (HGNC) Comparison of Orthology Predictions (http://www.genenames.org/cgi-bin/hcop.pl), National Center for Biotechnology Information (NCBI; http://www.ncbi.nlm.nih.gov/sites/entrez?db=pubmed), SOURCE (http://source.stanford.edu/cgi-bin/source/sourceSearch), EGO and TOGA using ortholog group number, gene name, TIGR accession number, Entrez gene IDs, Gene Accession number, Unigene cluster ID and Locus Link ID. JMP Statistical Discovery Software (http://www.jmp.com/) was used to generate a master data base containing all relevant probe specific annotation. The final ortholog database aggregating seven microarray platforms linked to 39,791 of 54,681 unique probes on the U133 Plus 2.0 platform. The master database file used in meta-analysis is available as supplementary material in **Table S1**.

### Classification and grouping of chips

Individual gene chips were classified and grouped based on the strategy used to induce lung injury [33]:

1. No Mechanical Ventilation (NV) – spontaneous breathing or sham animals (surgical procedures but no lung injury)
2. Inflammatory lung injury alone (Inf) – treatment with lipopolysaccharide (LPS, inhaled or intravenous) or hydrochloric acid (acid aspiration, AA) alone
3. One-hit (MV injury alone): injurious MV (HV, high tidal volume ≥15 ml/kg and/or b>1) or protective MV (LV, low tidal volume ≤12 ml/kg and/or b = 1)
4. Two-hit (inflammatory and MV injury): combined inflammatory and ventilation injury. For all studied using two-hit models the first hit was treatment with LPS or AA and the second was MV (HV or LV).

**Table 2** shows how chips were grouped into injury phenotypes of interest for the meta-analysis comparisons.

### Meta-analysis procedure

We used a random effects model to interpret changes in gene expression when comparing groups of chips in the experimental versus the control samples. The effect size was measured using the standardized mean difference for each gene in each individual experiment. We integrated the effect size across experiments using the random effect model. This allows for both within-experiment sampling error (variance) and between-studies variation to be included in the assessment of the uncertainty (confidence interval) of the results of a meta-analysis, thus selecting for genes with consistent patterns of expression across species, platforms and studies. A test statistic to evaluate the treatment effect is represented by the *z* score. The statistical significance of a change in gene expression is provided by calculating the p-value corresponding to this *z*-statistic, and then estimating the false discovery rates (FDR) for each significance level. This takes into account the number of tests performed and corrects for multiple comparisons. We considered ESTs with an FDR≤0.1 to be significantly altered.

We measured effect size 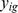 for gene *g* (assume gene and probe set are interchangeable terms here) in individual experiment *i* using the standardized mean difference [46], given by
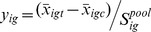
(1)where 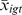 and 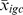 are the sample means of gene expression values for gene *g* in group t (e.g. treatment) and group c (e.g. control) of experiment *i*, respectively. 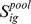 is the pooled standard deviation. The estimated variance 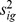 of the unbiased effect size 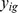 is given by [46]:
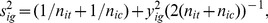
(2)For a experiment with 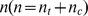 samples, an approximately unbiased estimate of 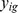 is given by 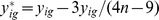.

To integrative analysis of effect sizes across studies using the random effects model, we suppose the effect size 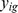 is estimated for gene *g* in experiment i (see **Table 1**), *i = 1,…*, 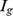. 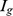 represents the number of experiments for the gene g. 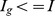, here I is the total number of experiments used in our meta-analysis (It should be noted that a probe set g on the U133 Plus 2.0 platform may be found in different number of experiments). We follow Choi et al. [27] to place the estimated 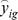 into a hierarchical model and to test for differences between groups:
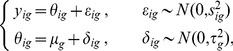
(3)where 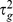 is the between-experiment variability of gene *g*, 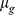 means the average measure of differential expression across the *I* experiments for gene *g*, which can be also called as mean effect size (MSE). Here, 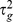 and 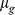 are gene-specific while 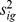 and 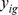 are gene and experiment-specific. 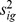 is the effect size variance of gene g, measuring the sampling error for the 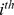 experiment. Using a random effects model [27], the meta-analysis estimate for 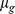 can be calculated as:
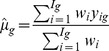
(4)where the weights are given by 
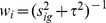
 and 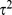 is the between-experiment) variability. The variance of this estimator is obtained by
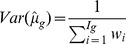
(5)A test statistic to evaluate the treatment effect of gene *g* across all *I* experiments can then be computed as
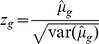
(6)We evaluated the statistical significance of a gene *g* by calculating the p-value corresponding to the z-statistic, and then estimated the false discovery rates (FDR) for each significance level, to take into account the number of tests performed [47]–[48].

### Validation of genes identified by meta-analysis

#### Validation using External Microarray Data Sets

Independent datasets from one mouse and two human in-vitro cell-based studies were used to validate the results from our microarray meta-analysis (**Table 7**). (a) Animal Model of HV induced lung injury: The data was downloaded from GEO (GSE11434), which compared HV ventilation versus NV. Overall design mice were anesthetized with isoflurane followed by ketamine/xylaxine. Saline (0.25 ml) was given every hour ip. A tracheotomy tube was placed and the mice were ventilated with an initial peak airway pressure of 20 cmH2O approximating a tidal volume of 20 ml/kg and without end-expiratory pressure. Ventilation was continued for 3 h. Control mice were treated identically, but were not mechanically ventilated (i.e. breathed spontaneously). There were 5 biological replicates in each group. (b) Human cyclic cell stretch data: First, human Bronchial Epithelial Cells (Beas-B2, GSE16650) cells grown on silicon elastic plates coated with Type I collagen (Flexercell International, McKeesport, PA) were exposed to six regiments for 4 h: 1) control (control); 2) mechanical stretch (25 PKa, 30 cycles per min (stretch); 3) LPS (1 mcg/ml, LPS); 4) TNF-α (20 ng/ml, TNF); 5) mechanical stretch plus LPS (LPS+S) and 6) mechanical stretch plus TNF-α (TNF+S). Total RNA (duplicate experiments) was extracted using TRIZOL reagent (as per manufactures specifications) and purified using Qiagen mRNA purification Kit (as per manufacturers specifications). mRNA was hybridized to Affymetrix Human U133plus2.0 chips [29]. Second, identical experiments carried out using primary Small Airway Epithelial Cells (SAEC, CC-2547, Human_UP in **Table 7**) purchased from Clonetics (/www.lonzabio.com). (c) Human transplant dataset: microarray data from fifty human lung transplant patients was obtained from GSE8021. In this study, total RNA from 50 donor lung samples were divided into two groups - those that developed PGD after transplantation (PGD positive) and those that did not (PGD negative). PGD was defined as T0 Grade III dysfunction according to International Society for Heart and Lung Transplantation criteria, that is, a ratio (referred to as the P/F ratio) of partial pressure of arterial oxygen (PaO2) to fraction of inspired oxygen (FiO2) less than 200 in the first arterial blood gas in the intensive care unit after transplantation (generally 4–6 hours after actual reperfusion). Total RNA from lungs were hybridized to Affymetrix GeneChip Human Genome U133A 2.0 Array. Normalized intensity values for individual gene probe sets were matched to our ortholog ID file. We ranked genes identified in the one- or two-hit model meta-analysis by adjusted p-value and empirically used the top 20 genes to classify the external data sets. Unsupervised hierarchical clustering was used to classify samples.

#### Prospective validation in rat two-hit model of lung injury

All experiments were approved by the Institutional Animal Care and Use Committee at Saint Michael's Hospital and performed in compliance with the Principles of Laboratory Care formulated by the Canadian Council and Animal Care. Male Sprague Dawley rats (n = 60; 300 to 350 g; Charles River Inc, Montreal, Quebec, Canada) were randomized to pneumonia by receiving intratracheal instillation with *Klebsiella pneumoniae* or equal volume saline. After 24 hrs rats were subsequently re-randomized to receive MV with either LV 6 ml/kg and 5 cm H2O of PEEP or HV 12 ml/kg without PEEP for 3 h as previously described13–15. Rats were anesthetized with ketamine hydrochloride (80 mg/kg, Ayerst Veterinary Laboratories, Guelph, Canada) and xylazine (8 mg/kg, Bayer, Toronto, Canada) administered intraperitoneally (IP). After tracheostomy a 14-gauge catheter was inserted into the trachea. The right carotid artery was cannulated with a 24-gauge angiocath (Becton Dickinson, Franklin Lakes, NJ) for measuring MAP, blood withdrawal and resuscitation. The tail vein was catheterized with a 22-gauge Angiocath (Becton Dickinson) for continuing infusion of ketamine hydrochloride (20 mg/kg/h), xylazine (4 mg/kg/h) and pancuronium (0.3 mg/kg/h). At the completion of the experiment rats were sacrificed, lung tissues were snap frozen. Total RNA was extracted for qRT-PCR. Expression of selected gene(s) was normalized to glyceraldehyde-3-phosphate dehydrogenase (GAPDH), 18S ribosomal subunit (18S) and beta-actin (β-actin).

### Classification Algorithms

The misclassification-penalized posterior (MiPP) [31] classifier was used to classify human lung injury samples into specific injury phenotypes based on results from the meta-analysis. MiPP uses a posterior probability of correct classification to judge the likelihood that a sample belongs in a given class. To build the classifier, MiPP sequentially selects genes one at a time using Linear Discriminant Analysis (LDA) to maximize the MiPP score. It adjusts the MiPP score by adding the posterior probabilities for classification of each sample in the training set and subtracting by 1 for each misclassification. Thus, for each number of genes, the MiPP algorithm chooses the optimal set of genes and builds a predictor based on the training dataset.

The k-top scoring pair (k-TSP) method [32] is an extension of top scoring pair (TSPs) algorithm [49], which was also used to classify the 50 human samples into one of the two classes (PGF and Good). The algorithm is a rank-based method and uses k-pair of genes for classification. The k is decided by five-fold cross-validation. It is more robust to variation in technical factors or normalization than classifiers based on expression levels of individual genes. We used *ktspair* R package (http://cran.r-project.org/web/packages/TSP/index.html) to perform the analysis. The ROC curves were generated based on the offset method implemented in the package [32].

## Supporting Information

Figure S1 **Heat maps of estimated effect sizes of top genes from one-hit model (a) and two-hit model (b).** For the top 20 genes shown in **Figure 4a** and **Figure 4b**, we showed heat maps of the estimated effect sizes of 8 of the 20 genes from one-hit model in 8 studies (a) (note: only 8 of the 20 genes have expression profiles in multiple platforms and species) and of 15 genes from two-hit model in 3 studies (b). The maps indicate the estimated effect sizes of the same gene have the same direction across multiple platforms and species.
(TIF)

Figure S2 **Distribution of clustering accuracies based on 1000 random gene sets.** (a) 20 random genes were selected from independent animal lung injury microarray experiment (GSE11434, **Table 7**), which were used to cluster gene expression data of animal lung injury samples in one-hit model (NV or LV vs. HV) and clustering accuracy was calculated. The procedure was repeated 1000 times. (b) 20 random genes were selected from independent human microarray experiment (GSE16650, **Table 7**), which were used to cluster gene expression data of human bronchial epithelial distal airway small cells (BEAS2b) samples in one-hit model and clustering accuracy was calculated. The procedure was repeated 1000 times.
(TIF)

Figure S3 **Clustering of gene expression data of independent animal and human samples based on top genes selected from individual study.** Top 20 genes were selected from one-hit model in an individual study (GSE2411, **Table 2**; The reason for selecting this study is that it has the largest sample size) based on effect size. These genes were used to classify lung injury samples from an independent microarray experiment, not included in the meta-analysis (GSE11434, **Table 7**) (a) and an independent human bronchial epithelial distal airway small cells (BEAS2b) microarray experiment (GSE16650, **Table 7**) (b). The top 20 genes from the differential analysis of the individual study used in meta-analysis identified group assignment correctly for all independent animal samples (a), but can not identify group assignment correctly for independent human samples (b).
(TIF)

Table S1 **Orthologue Data Base.**
(TXT)

Table S2 **Differentially expressed genes selected in one-hit model at FDR< = 0.1.**
(CSV)

Table S3 **Differentially expressed genes selected in two-hit model at FDR< = 0.1.**
(CSV)
