# Supplementary figures and images for: Microarray Meta-Analysis Identifies Acute Lung Injury Biomarkers in Donor Lungs That Predict Development of Primary Graft Failure in Recipients
Source: PLoS One. 2012 Oct 12;7(10):e45506. doi: 10.1371/journal.pone.0045506 (PMC3470558; doi:10.1371/journal.pone.0045506)

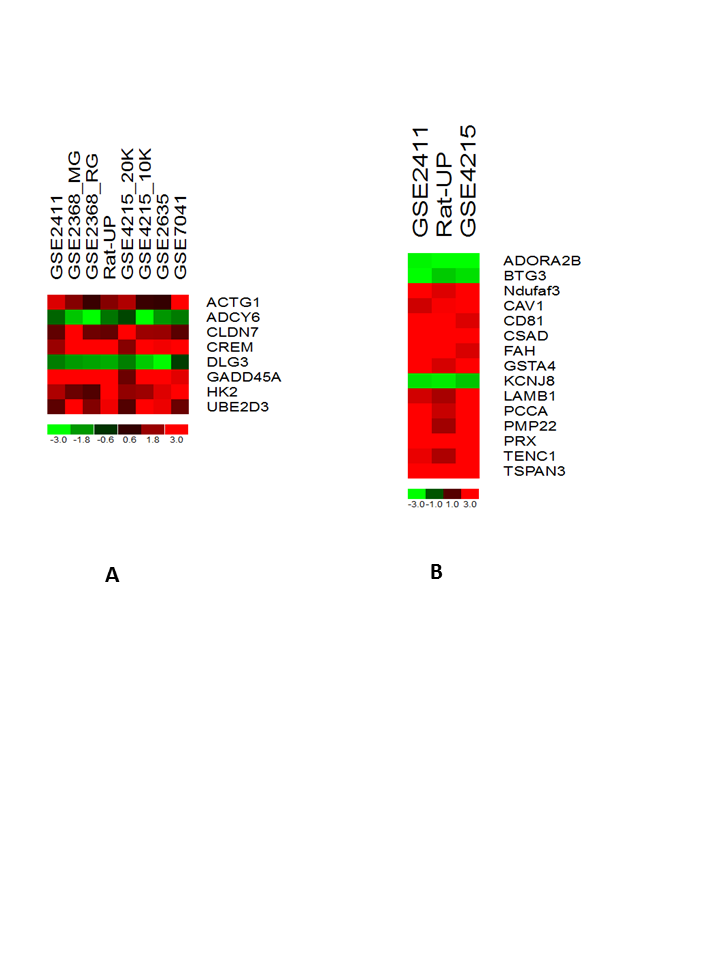

Supplement: Figure S1 — Heat maps of estimated effect sizes of top genes from one-hit model (a) and two-hit model (b). For the top 20 genes shown in Figure 4a and Figure 4b , we showed heat maps of the estimated effect sizes of 8 of the 20 genes from one-hit model in 8 studies (a) (note: only 8 of the 20 genes have expression profiles in multiple platforms and species) and of 15 genes from two-hit model in 3 studies (b). The maps indicate the estimated effect sizes of the same gene have the same direction across multiple platforms and species. (TIF) [file pone.0045506.s001.tif]

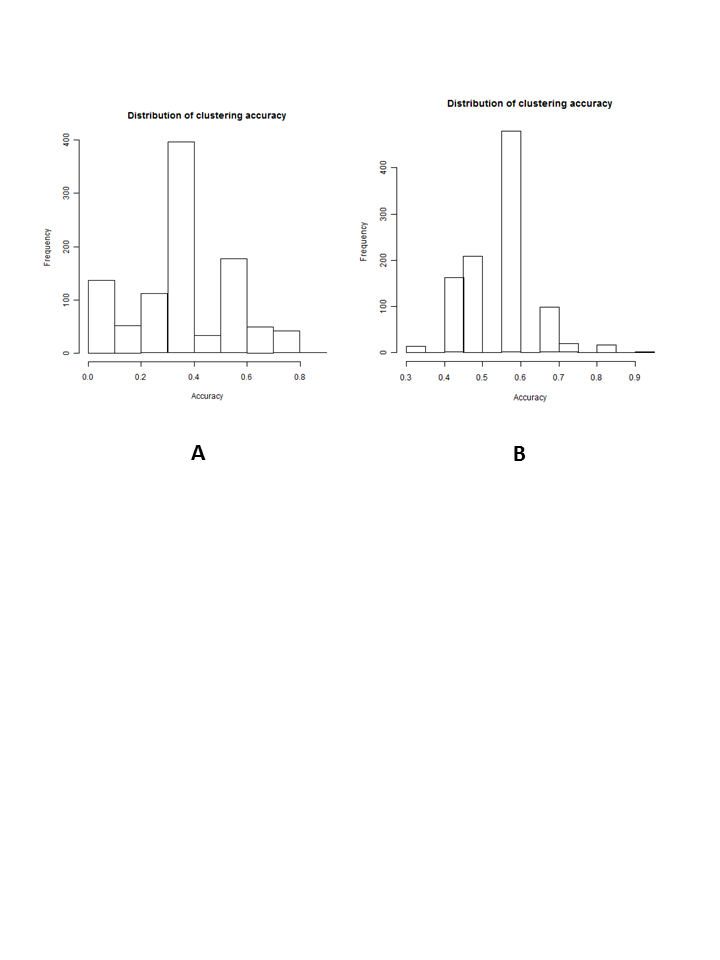

Supplement: Figure S2 — Distribution of clustering accuracies based on 1000 random gene sets. (a) 20 random genes were selected from independent animal lung injury microarray experiment (GSE11434, Table 7 ), which were used to cluster gene expression data of animal lung injury samples in one-hit model (NV or LV vs. HV) and clustering accuracy was calculated. The procedure was repeated 1000 times. (b) 20 random genes were selected from independent human microarray experiment (GSE16650, Table 7 ), which were used to cluster gene expression data of human bronchial epithelial distal airway small cells (BEAS2b) samples in one-hit model and clustering accuracy was calculated. The procedure was repeated 1000 times. (TIF) [file pone.0045506.s002.tif]

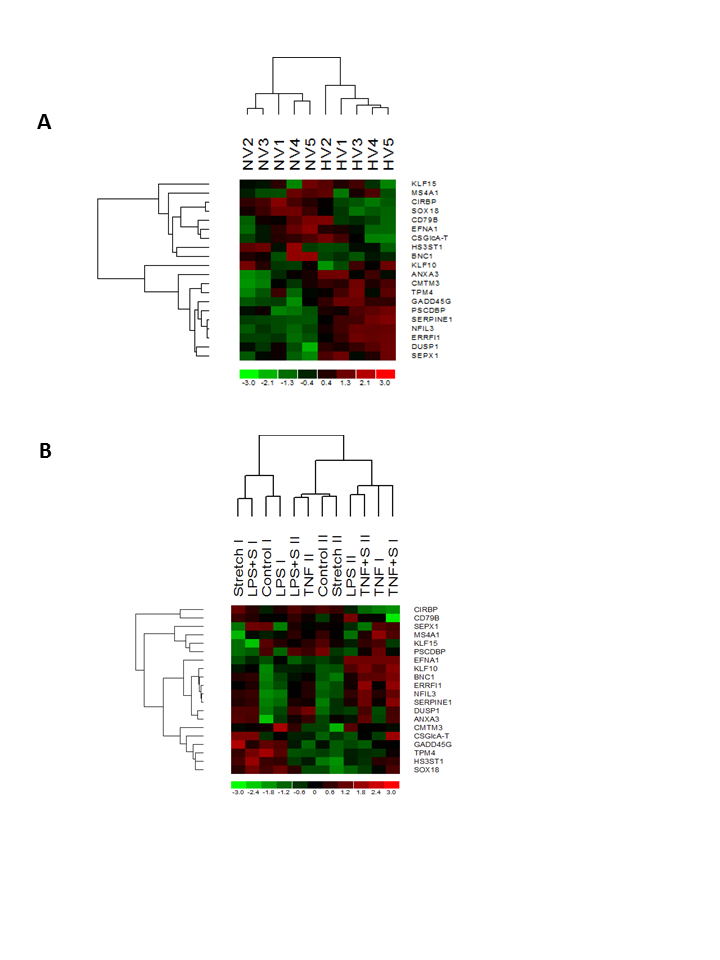

Supplement: Figure S3 — Clustering of gene expression data of independent animal and human samples based on top genes selected from individual study. Top 20 genes were selected from one-hit model in an individual study (GSE2411, Table 2 ; The reason for selecting this study is that it has the largest sample size) based on effect size. These genes were used to classify lung injury samples from an independent microarray experiment, not included in the meta-analysis (GSE11434, Table 7 ) (a) and an independent human bronchial epithelial distal airway small cells (BEAS2b) microarray experiment (GSE16650, Table 7 ) (b). The top 20 genes from the differential analysis of the individual study used in meta-analysis identified group assignment correctly for all independent animal samples (a), but can not identify group assignment correctly for independent human samples (b). (TIF) [file pone.0045506.s003.tif]
